# Induction of the intrinsic apoptotic pathway via a new antimitotic agent in an esophageal carcinoma cell line

**DOI:** 10.1186/2045-3701-4-68

**Published:** 2014-11-20

**Authors:** Elize Wolmarans, Katherine Sippel, Robert McKenna, Annie Joubert

**Affiliations:** Department of Physiology, University of Pretoria, Pretoria, South Africa; Department of Biochemistry and Molecular Biology, Baylor College of Medicine, Houston, Texas USA; McKnight Institute, University of Florida, Gainesville, Florida USA

**Keywords:** 2-Ethyl-3-*O*-sulphamoyl-estra-1,3,5(10)16-tetraene, Esophageal carcinoma, Intrinsic apoptotic pathway

## Abstract

**Background:**

2-Ethyl-3-*O*-sulphamoyl-estra-1,3,5(10)16-tetraene (ESE-16) is a unique, *in silico*-designed compound with possible anticancer properties, which were identified in our laboratory. This compound is capable of interfering with microtubule dynamics and is believed to have potential carbonic anhydrase IX inhibiting activity.

In this study, it was investigated whether ESE-16 is capable of inducing apoptosis *in vitro* in the esophageal carcinoma SNO cell line via the intrinsic pathway at a concentration of 0.2 μM with an exposure time of 24 hours.

**Results:**

Qualitative results were obtained via light microscopy, transmission electron microscopy and confocal microscopy. Results showed hallmarks of apoptosis in the ESE-16-treated cells. In addition, data revealed an increase in the number of ESE-16-treated cells blocked in metaphase. Cell death via apoptosis in the ESE-16-treated cells was confirmed by studying the internal ultrastructure of the cells via transmission electron microscopy, while confocal microscopy revealed abnormal spindle formation and condensed chromatin in ESE-16-treated cells, thus confirming metaphase block.

Quantitative results were obtained via flow cytometry and spectrophotometry. Cell death via apoptosis in ESE-16-treated cells was quantitatively confirmed by the Annexin V-FITC apoptosis detection assay. Flow cytometry and spectrophotometry revealed dissipation of mitochondrial membrane potential and an increase in superoxide levels in the ESE-16-treated cells when compared to the relevant controls. Both initiator caspase 9 and effector caspase 3 activities were increased, which demonstrates that ESE-16 causes cell death in a caspase-dependent manner.

**Conclusions:**

This was the first *in vitro* study conducted to investigate the action mechanism of ESE-16 on an esophageal carcinoma cell line. The results provided valuable information on the action mechanism of this potential anticancer agent. It can be concluded that the novel *in silico*-designed compound exerts an anti-proliferative effect on the esophageal carcinoma SNO cell line by disrupting microtubule function resulting in metaphase block. This culminates in apoptotic cell death via the intrinsic apoptotic pathway. This research provided cellular targets warranting *in vivo* assessment of ESE-16’s potential as an anticancer agent.

## Background

Microtubule-interfering drugs (MIDs) are one of the most promising classes of cancer chemotherapeutic drugs available [1–3]. MIDs target the cell cycle by binding to and interfering with the microtubule machinery, thereby inhibiting the normal function of the mitotic spindle and preventing hyperproliferation of cancer cells [1, 4–6]. Owing to the therapeutic success of MIDs, intense search and development for new microtubule-targeting compounds are being conducted by pharmaceutical companies [2].

**Figure 1:**
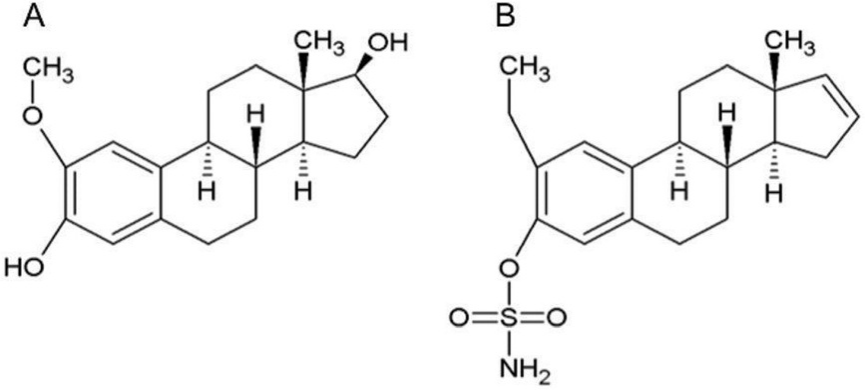
**Structural comparison between** **(A)** **2ME and** **(B)** **ESE-16.** When the two chemical structures are compared, an exchange of a sulphamoylated group for a hydroxyl group at position 3 and the removal of a hydroxyl group at position 17 on the ESE-16 compound are noticed. The sulphamoylated group increases the bioavailability of the compound [8, 13, 20, 28, 29], while the modifications at position 3 and −17 increases the anticancer potency and provides a prolonged half-life [6, 11, 13, 22, 27, 28].

2-Methoxyestradiol (2ME) (Figure 1A) is an endogenous metabolite derived from 17β-estradiol [6–12] with antiproliferative, anti-angiogenic and pro-apoptotic characteristics *in vitro* and *in vivo*[6–8, 13, 14] independent of estrogen receptors [6, 12, 15, 16]. 2ME’s major target is the microtubule skeleton [6, 7, 11, 12, 14, 16]. The compound influences the spindle assembly checkpoint (SAC) by interacting with the colchicine binding site situated between the α- and β-dimers of the tubulin protein [8, 9, 14, 17, 18]. This interaction causes abnormal spindle formation and activation of the spindle checkpoint, which leads to metaphase arrest, inhibition of further cell proliferation and the induction of cell death [6, 10, 11, 19].

2ME, however, has been found to have low bioavailability in the human body as a result of rapid metabolic degradation by the enzyme 17β-hydroxysteroid dehydrogenase type 2 in the gastrointestinal tract and liver [8, 13, 20–22]. The compound is registered as “Panzem” by Entremed Inc. (Rockville, Maryland, USA) and is currently undergoing clinical trials with an innovative nanocrystal dispersion (NCD) drug delivery system which may possibly overcome the rapid biodegradation problem [23–26].

Researchers have attempted to create analogues of 2ME with improved bioavailability and potency. The analogue being investigated in this research was the latest *in silico*-designed 2ME derivative: 2-ethyl-3-*O*-sulphamoyl-estra-1,3,5(10)16-tetraene (ESE-16). ESE-16 (Figure 1B) was developed when potential carbonic anhydrase IX (CAIX) inhibitors, capable of interfering with microtubule dynamics were identified in our laboratory with the use of bioinformatics software [8].

When the chemical structures of ESE-16 and 2ME are compared (Figure 1), an exchange of a sulphamoylated group for a hydroxyl group at position 3 and the removal of a hydroxyl group at position 17 on the ESE-16 compound are noticed. Previous studies have shown sulphamoylated analogues with modifications on the 3- and 17-position of the molecule revealed significant anticancer potency and prolonged half-life and bioavailability [6, 11, 13, 22, 27, 28]. Increased bioavailabilty is due to the ability of the sulfamoylated derivatives to reversibly bind to carbonic anhydrase II (CAII) found in red blood cells, thereby enabling them to journey through the liver without undergoing presystemic metabolism [8, 13, 20, 28, 29].

Our laboratory has demonstrated the anti-proliferative action of ESE-16 in a variety of cell lines: the tumorigenic human epithelial cervical HeLa cell line, MCF-7 breast cancer cell line, esophageal carcinoma SNO cell line, metastatic MDA-MB-231 breast cancer cell line and the non-tumorigenic MCF-12A cell line [8, 29–32].

Studies have also revealed ESE-16 to be more potent than its source compound, 2ME [8]. Research has shown that 2ME exerts antiproliferative effects at concentrations between 1-2 μM, whereas ESE-16 has shown antiproliferative activity at nanomolar values, with a GI_50_ value of 180-220nM [8].

The exact action mechanism of ESE-16, however, still remains to be elucidated. This *in vitro* study was the first to investigate the action mechanism of ESE-16 on an esophageal carcinoma cell line.

It was hypothesized that ESE-16 uses the intrinsic apoptotic pathway as an action mechanism to cause cell death. In the hypothesized chain of events the compound binds to the microtubules of the esophageal carcinoma cells, causing the activation of the SAC and subsequent metaphase arrest. This leads to increased reactive oxygen species (ROS) production, mitochondrial membrane potential (∆Ψm) dissipation, degradation of the mitochondrial membrane and the release of cytochrome *c*. Cytochrome *c* then binds with apoptotic protease activating factor 1 (Apaf-1) to form the apoptosome, which activates the initiator caspase 9. Caspase 9 activates the effector caspase 3, which then leads to the cell undergoing apoptosis.

The results provided valuable information on the action mechanism of this potential anticancer agent. It can be concluded that the novel *in silico*-designed compound exerts an anti-proliferative effect on the esophageal carcinoma SNO cell line by disrupting microtubule function, resulting in metaphase block. This culminates in apoptotic cell death via the intrinsic apoptotic pathway.

## Results

### Haematoxylin and eosin staining show morphological changes in the ESE-16-treated SNO cells

**Figure 2:**
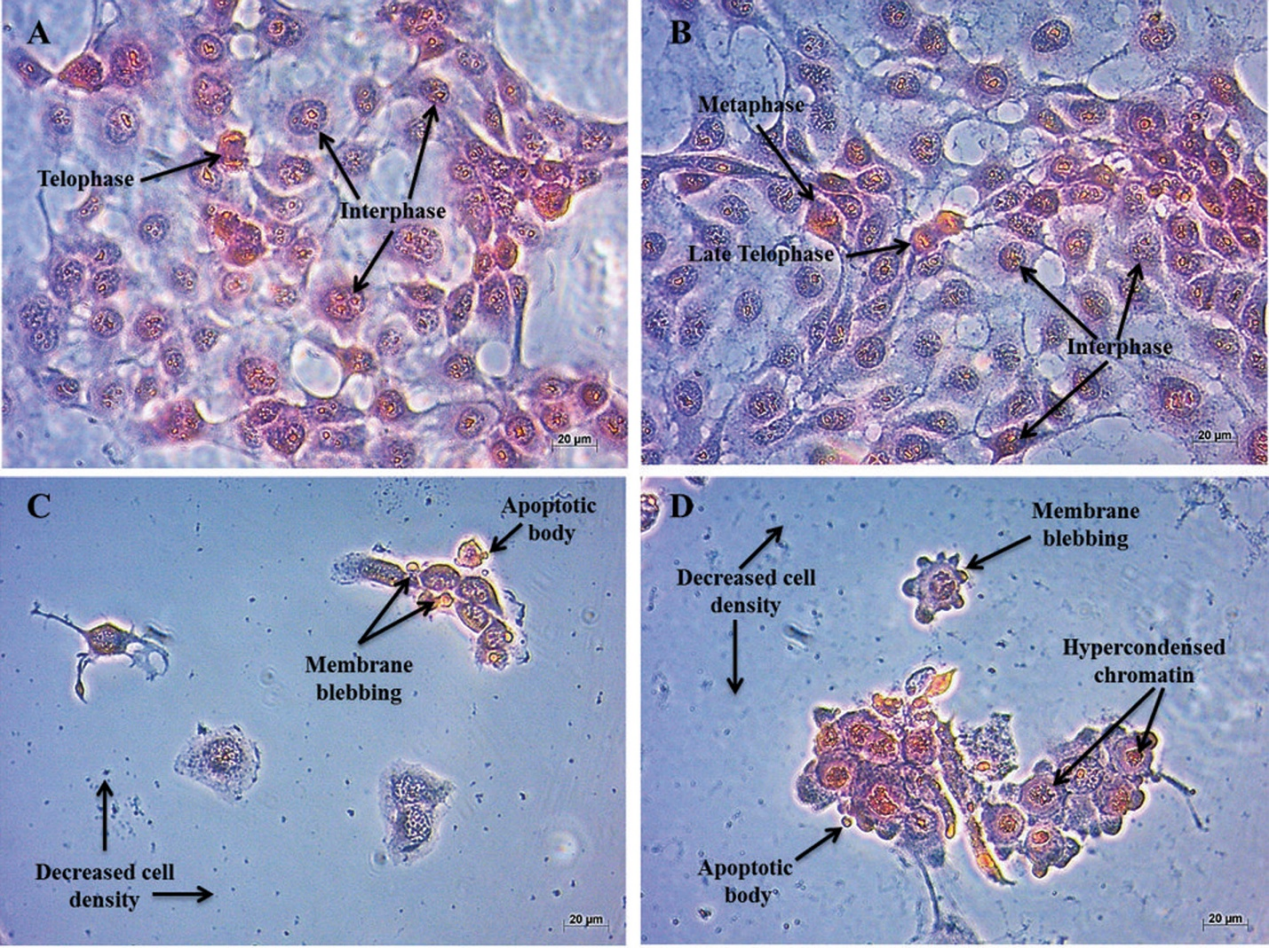
**Haematoxylin and eosin staining images revealing morphological changes in the nuclear and cytoplasmic components in SNO cells. (A)** Cells propogated in medium only, **(B)** cells exposed to dimethyl sulphoxide (DMSO) – vehicle control, **(C)** cells exposed to actinomycin D – positive control and **(D)** cells exposed to ESE-16 at a concentration of 0.2 μM. Cells propogated in medium and the vehicle-treated cells showed normal cell morphology, with the majority of the cells being in interphase. The positive control for apoptosis and the ESE-16 treated cells showed a decrease in cell density and morphological characteristics of apoptosis such as membrane blebbing and apoptotic bodies. The ESE-16 treated cells also showed an increase in the number of round cells with hypercondensed chromatin indicative of a metaphase block (Magnification: ×20).

Haematoxylin and eosin (H&E) staining allows for the quantitative comparison of the morphological characteristics of the cytoplasm and nuclear components of the cells [10]. The staining was used to visualize morphological changes in SNO cells after exposure to ESE-16 and the appropriate controls. Cells propogated in medium (Figure 2A) and the vehicle control (Figure 2B) showed normal cell morphology with the majority of the cells being in interphase. The positive control for apoptosis (Figure 2C) and ESE-16-treated cells (Figure 2D) showed a decrease in cell density and morphological characteristics of apoptosis such as membrane blebbing and apoptotic bodies. ESE-16-treated cells also showed an increase in the number of round cells with hypercondensed chromatin indicative of metaphase block.

In order to obtain quantitative data from this morphological study, mitotic indices were determined (Table 1) [10]. One thousand cells were counted on each slide of the biological replicates and divided into their different phases [10, 33]. Identification of cells in interphase, the different mitotic phases and cells undergoing apoptosis were done based on their cellular and nuclear morphology [34]. Cells that could not be categorized as a result of excessive fragmentation, unusual nuclear morphology or a lack of clear nuclear material were defined and counted as abnormal [11]. The data is expressed as percentages of cells in each phase. Mitotic indices revealed a significant increase, with a *P*-value of 0.0003^*^, in the percentage of cells in metaphase in the ESE-16-treated samples when compared to controls. ESE-16-treated cells also showed a significant increase in the percentage of cells undergoing apoptosis when compared to the appropriate controls (*P*-value of 0.0006)^*^.

**Table 1:** **Average percentage of cells in interphase**, **various stages of mitosis and cells displaying apoptotic morphology**

	Interphase	Prophase	Metaphase	Anaphase	Telophase	Apoptosis	Abnormal
**Medium**	96.1%	0.6%	1.5%	0.4%	0.6%	0.5%	0.4%
**DMSO**	97.5%	0.2%	1.3%	0.2%	0.6%	0.2%	0.4%
**Actinomycin D**	60.2%	0.3%	2.5%	0%	0.5%	33.8%	2.3%
**ESE**-**16**	27.3%	0.7%	46.0%^*^	0.2%	0.4%	21.4%^*^	4.2%

Mitotic indices revealed a significant increase, with a P-value of 0.0003*, in the percentage of cells in metaphase in the ESE-16-treated samples (46.0%) when compared to the vehicle control (1.3%). ESE-16-treated cells also showed a significant increase (P-value of 0.0006*) in the percentage of cells undergoing apoptosis (21.4%) when compared to the vehicle control (0.2%).

### Transmission electron microscopy shows changes in the internal ultrastructure of the ESE-16-treated SNO cells

**Figure 3:**
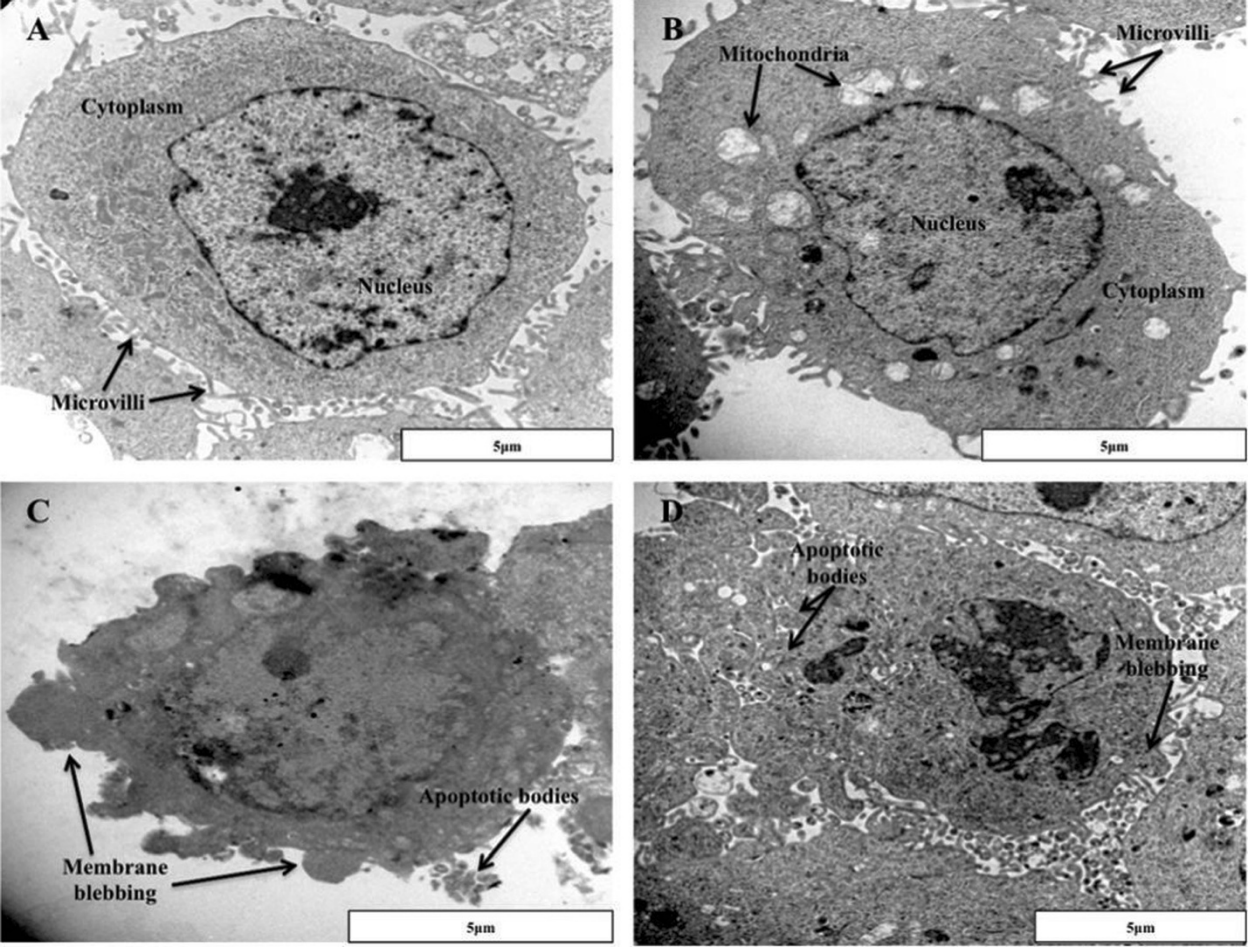
**Transmission electron microscopy images providing information on the internal ultrastructure of SNO cells propogated in medium only and SNO cells treated with DMSO. (A)** Cells propogated in medium only, **(B)** cells exposed to DMSO – vehicle control, **(C)** cells exposed to actinomycin D – positive control and **(D)** cells exposed to ESE-16 at a concentration of 0.2 μM. Both the cells propogated in medium and the vehicle control cells showed microvilli protruding from their cell membrane surface. The nuclear membrane is smoothly outlined and well-preserved cytoplasmic organelles are visible. The positive control for apoptosis showed loss of microvilli, membrane blebbing and the presence of apoptotic bodies. ESE-16-treated cells revealed a decrease in nuclear membrane definition, membrane blebbling and apoptotic body formation. Scale bar: 5 μm.

Transmission electron microscopy (TEM) was used to study the internal ultrastructure of the SNO cells after exposure to ESE-16 and the appropriate controls. Results revealed normal infrastructure of cells propogated in medium (Figure 3A) and in the vehicle control (Figure 3B). Both the cells propogated in medium and the vehicle control revealed microvilli protruding from their cell membrane surface, a smoothly outlined nuclear membrane and well-preserved cytoplasmic organelles. The positive control for apoptosis (Figure 3C) showed loss of microvilli, membrane blebbing and the presence of apoptotic bodies. An ESE-16-treated cell image (Figure 3D) revealed a decrease in nuclear membrane definition, membrane blebbling and apoptotic body formation.

### Confocal microscopy reveals the effect ESE-16 has on the microtubule architecture of the SNO cells

**Figure 4:**
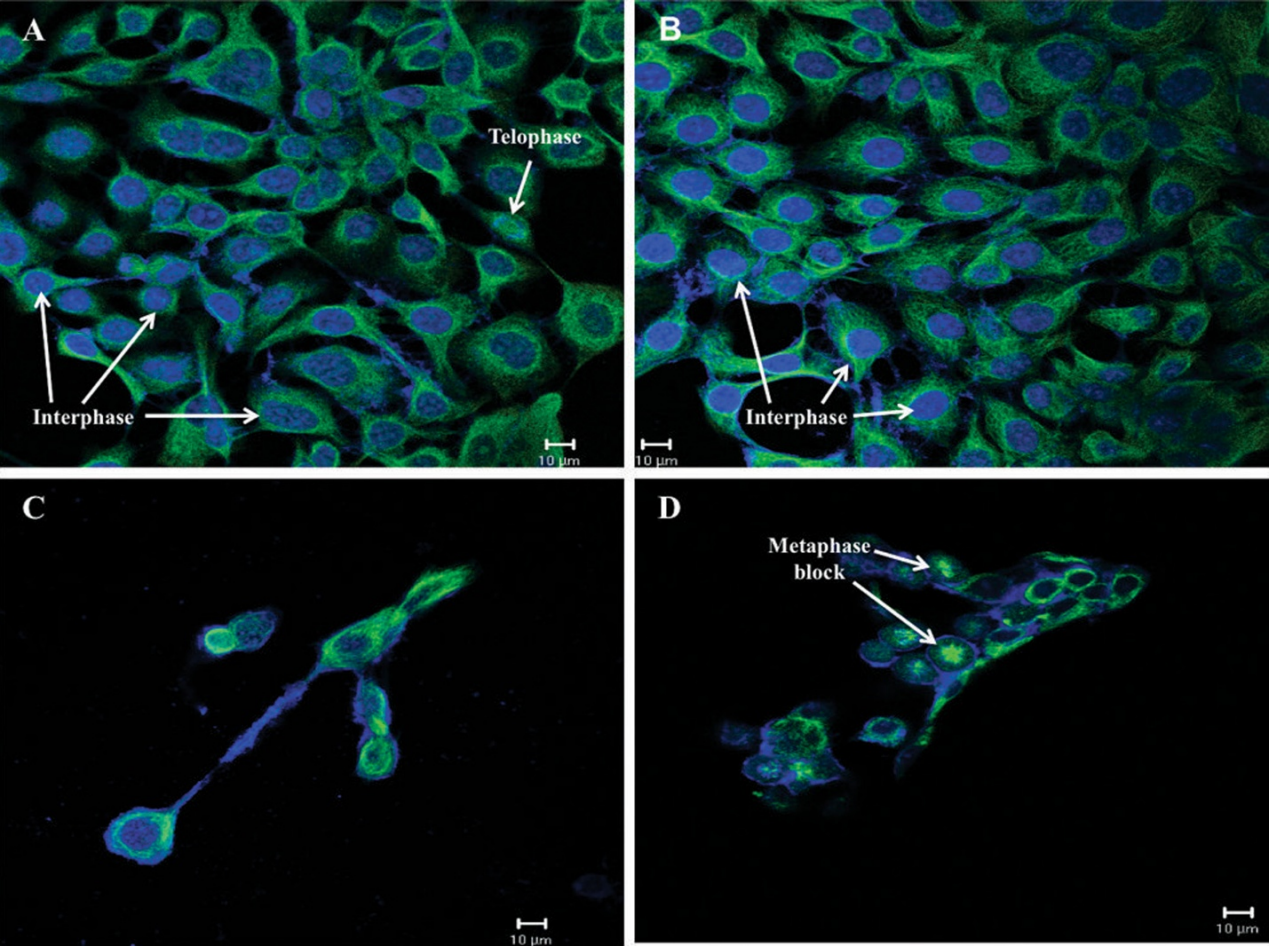
**Confocal microscopy images of the microtubule architecture of SNO cells with the use of anti-α** **tubulin antibodies and nuclear stain 4**′,**6**-**diamidino**-**2**-**phenylindole. (A)** Cells propogated in medium only, **(B)** cells exposed to DMSO – vehicle control, **(C)** cells exposed to actinomycin D – positive control and **(D)** cells exposed to ESE-16 at a concentration of 0.2 μM. The α-tubulin of the cells were stained green by a FITC-conjugate diluent while the nuclei were stained blue by DAPI to provide contrast to the green fluorescence. Cells propogated in medium and vehicle control cells showed normal microtubule architecture. The positive control showed a decrease in cell density and revealed shrunken cells. ESE-16-treated cells showed a decrease in cell density and abnormal spindle formation when compared to the appropriate controls. The abnormal spindle formation is indicative of metaphase block. Scale bar: 10 μm.

Confocal microscopy was used to determine the influence of ESE-16 on the cytoskeletal microtubule architecture of SNO cells after exposure to ESE-16 and the appropriate controls. Cells were stained with mouse monoclonal antibody against human α-tubulin and a secondary antibody, biotin-conjugated anti-mouse IgG 58 (Fab-specific, developed in goat) in a fluorescein isothiocyanate (FITC)-conjugate diluent, which stained the α-tubulin of the cells green. 4′,6-Diamidino-2-phenylindole (DAPI) was used to stain the nuclei of the cells blue to provide contrast to the green stain. Cells propogated in medium (Figure 4A) and vehicle control cells (Figure 4B) showed normal microtubule architecture. The positive control (Figure 4C) showed a decrease in cell density and revealed shrunken cells. The ESE-16-treated cells (Figure 4D) also showed a decrease in cell density and revealed abnormal spindle formation, indicating cells being blocked in metaphase.

### Apoptosis induction in the ESE-16-treated SNO cells

**Figure 5:**
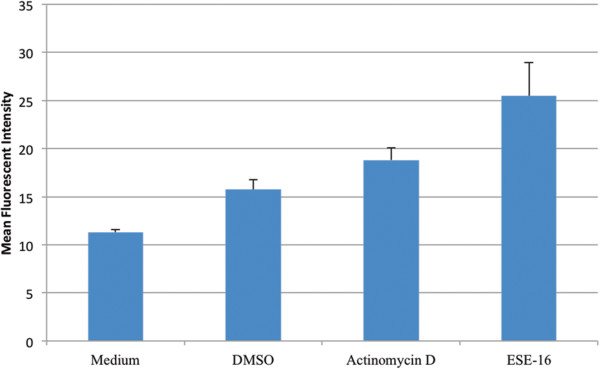
**Bar graph showing the average mean fluorescent intensity increase in the ESE**-**16**-**treated cells compared to the appropriate controls illustrating an increase in phosphatidylserine externalization.** This bar graph represents the average MFI of all three repeats done. Cells propogated in medium had an average MFI of 11.31, the vehicle control had an average MFI of 15.74, the positive control had an average MFI of 18.82 while the ESE-16-treated cells an average MFI of 25.50. The increased MFI indicates an increase in PS externalization which is an early apoptotic indicator [35].

Annexin V-FITC, a well-known apoptosis-detecting assay, was used to confirm that apoptosis was taking place in the SNO cells after exposure to ESE-16 (Figure 5). Results revealed an average increase in the mean fluorescent intensity (MFI) in the ESE-16-treated cells (average of 25.50) when compared to the vehicle control with an average MFI of 11.32. The increase in MFI indicates an increase in phosphatidylserine (PS) externalization, which is an early apoptotic indicator [35]. The *P*-value obtained for this experiment, however, was 0.0614, which is statistically insignificant. The statistically insignificant increase may be due to the fact that PS externalization is an early sign of apoptosis and that, at the time of experiment termination, the cells may have already surpassed the early apoptotic stage. This may also explain the low MFI value of the positive control (average of 18.82).

### Decrease in mitochondrial membrane potential in ESE-16-treated SNO cells

The Mitotracker Kit was used to study the possible influence ESE-16 has on the ∆Ψm of the SNO cells. The mitochondria are labeled by a cationic dye named “5,5′,6,6’-tetrachloro-1,133’-tetra-ethylbenzimidazolyl-carbocyanine iodide”, which passively diffuses across the plasma membrane and accumulates in active mitochondria [36]. Reduction of the ∆Ψm is another feature of apoptosis that is due to the loss of the electrochemical gradient across the mitochondrial membrane. With the reduction of the ∆Ψm, the mitotracker dye cannot aggregate in the mitochondria and thus remains in the cytoplasm in its monomer form, generating green fluorescence [36].

**Figure 6:**
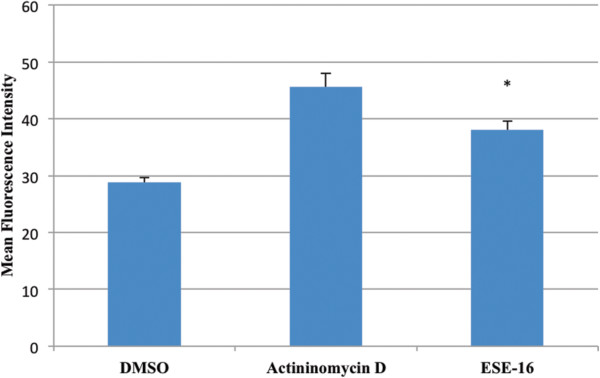
**Bar graph showing the average mean fluorescent intensity increase in the ESE**-**16**-**treated cells compared to the appropriate controls**, **illustrating a decrease in mitochondrial membrane potential.** This bar graph represents the average MFI of all three repeats done. The vehicle control had an average MFI of 28.79 while the postive control had an average MFI of 45.58 and the ESE-16-treated cells an average MFI of 37.995. The increase in the MFI seen in the ESE-16-treated cells is statistically significantly (*P*-value of 0.019)^*^ higher than that of the vehicle control indicating a decrease in ∆Ψm and possible mitochondrial membrane degradation.

Results obtained showed a statistcally significant (*P*-value of 0.019)^*^ increase in the MFI of green fluorescence in the ESE-16-treated cells when compared to the appropriate controls (Figure 6). These results demonstrate the ability of ESE-16 to cause a decrease in ∆Ψm. These findings confirm the qualitative and quantitative data obtained with regard to ESE-16 causing apoptosis at a concentration of 0.2 μM after an exposure time of 24 hours. The results also provide evidence of the degradation of the mitochondrial membrane, indicating apoptotic cell death via the intrinsic pathway.

### Increase in superoxide levels in ESE-16-treated SNO cells

**Figure 7:**
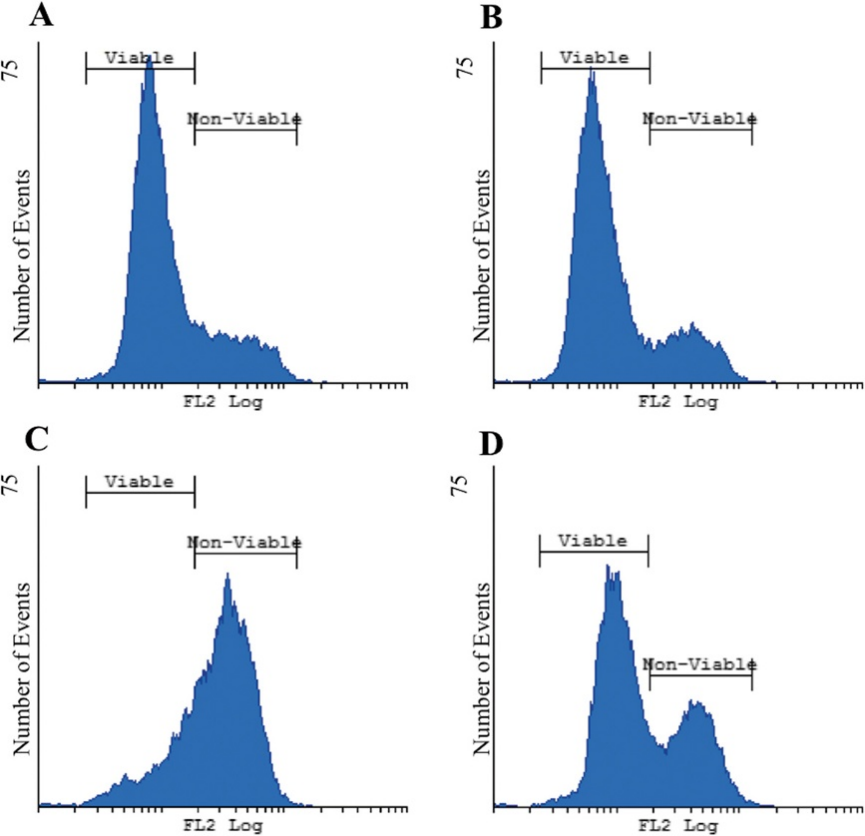
**Histograms illustrating superoxide levels in SNO cells after exposure to ESE**-**16 and the appropriate controls of a representative repeat. (A)** Cells propogated in medium only, **(B)** cells exposed to DMSO – vehicle control, **(C)** cells exposed to actinomycin D – positive control and **(D)** cells exposed to ESE-16 at a concentration of 0.2 μM. Viable cells represent the population of cells which showed little or no increase in fluorescence, thus no increase. The non-viable cells represent the population of cells with an increase in fluorescence thus indicating an increase in O_2_ ^−^ production. The ESE-16-treated cells showed 38.17% of its population to be non-viable compared to the 21.45% non-viable population of the vehicle control.

The accumulation of ROS may lead to oxidative damage to mitochondrial proteins and mitochondrial deoxyribonucleic acid (DNA), causing loss of electron transport, decrease in adenosine triphosphate (ATP) production and ∆Ψm dissipation [37–40]. Superoxide anion (O_2_ ^−^) can be regarded as the precursor for most ROS [40]. Thus, flow cytometry was used to measure O_2_ ^−^ levels in the SNO cells after exposure to ESE-16 and the various controls (Figure 7).

Viable cells represent the population of cells that showed little or no increase in fluorescence, thus no increase in O_2_^−^ production. Non-viable cells represent the population of cells with an increase in fluorescence, thus indicating an increase in O_2_ ^−^ production. Results revealed an increase in O_2_ ^−^ production in the ESE-16 treated cells with 38.17% of its population being non-viable when compared to the 21.45% non-viable population of the vehicle control (Table 2). Results indicate that ESE-16 does cause an increase in O_2_ ^−^ levels, which may lead to mitochondrial degradation and cell death. This confirms results obtained from studying the ∆Ψm.

**Table 2:** **Percentages of viable and non**-**viable cells of the representative repeat**, **illustrating superoxide levels in SNO cells after exposure to ESE**-**16 and the appropriate controls**

	Medium	DMSO	Actinomycin D	ESE-16
**Viable cells**	75.18%	77.90%	22.61%	60.11%
**Non**-**viable cells**	23.45%	21.05%	75.29%	38.17%

### Increase in caspase activity in the ESE-16-treated SNO cells

The activity of initiator caspase 9 and the effector caspase 3 in SNO cells after exposure to ESE-16 or the appropriate controls was studied via spectrophotometry. Results, showing the average ratio to medium, revealed an increase in both caspase 9 and caspase 3 activity in the ESE-16-treated cells when compared to the vehicle control.

**Figure 8:**
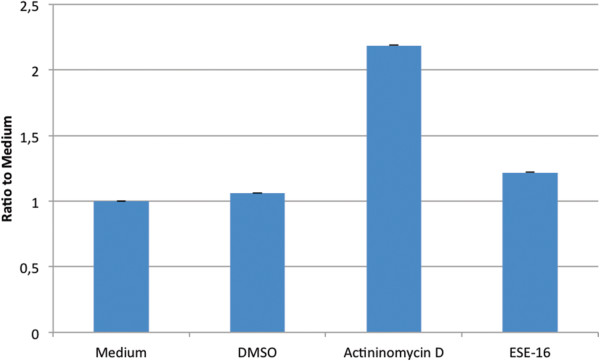
**Bar graph illustrating the ratio to medium of the initiator caspase 9 levels in SNO cells exposed to ESE**-**16 and various controls.** Results revealed a statistically insignificant increase (*P*-value of 0.238) in caspase 9 activity in the ESE-16-treated cells when compared to the vehicle control. The ESE-16-treated cells had a ratio to medium value of 1.2188, while the vehicle control had a ratio to medium value of 1.0625.

**Figure 9:**
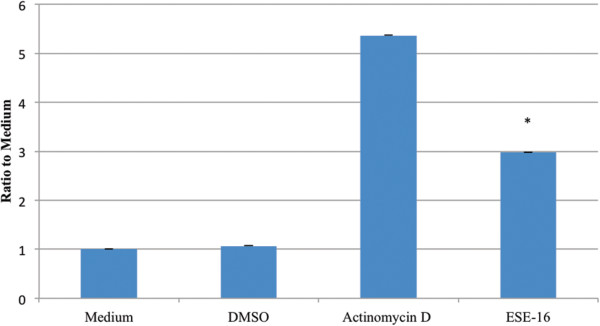
**Bar graph illustrating the ratio to medium of the effector caspase 3 levels in SNO cells exposed to ESE**-**16 and various controls.** Results revealed a statistically significant increase (*P*-value of 0.004)^*^ in caspase 3 activity in the ESE-16-treated cells when compared to the vehicle control. The ESE-16-treated cells had a ratio to medium value of 2.9772, while the vehicle control had a ratio to medium value of 1.0681.

The initiator caspase 9 results (Figure 8) showed that ESE-16-treated cells had a ratio to medium value of 1.2188, while the vehicle control had a value of 1.0625. This is not a statistically significant increase (*P*-value of 0.238) when compared to the difference in caspase 3 ratio to medium values. The effector caspase 3 results (Figure 9) showed ESE-16-treated cells had an average ratio to medium value of 2.9772 while the vehicle control had a value of 1.0681. This is a statistically significant increase, with a *P*-value of 0.004^*^.

The difference in the increase of activity of the two caspases may be explained by the fact that the effector caspase 3 had already, at the time of measurement, begun the degradation phase of apoptosis [35, 41], causing a decrease in caspase 9 levels. The caspase activity results show that ESE-16 causes the activation of caspases and reveals that ESE-16 causes cell death in a caspase-dependent manner.

## Discussion

ESE-16, a unique, *in silico*-designed compound was designed with the dual capability of interfering with microtubule dynamics [1] and inhibiting CAIX, which is over-expressed in a variety of tumours [8, 42]. These two targets were chosen because: 1) several studies have shown that inhibition of CAIX can lead to decreased invasiveness and may cause cell death under hypoxic conditions [42] and 2) by binding to microtubules there is interference during cell division, which may lead to cell death [1, 4–6].

In this study ESE-16 was shown to induce apoptosis *in vitro* in the esophageal carcinoma SNO cell line via the intrinsic pathway at a concentration of 0.2 μM with an exposure time of 24 hours. The concentration of 0.2 μM for ESE-16 was chosen since previous dose-dependent investigations conducted in our laboratory showed ESE-16 inhibiting cell proliferation to 50% from concentrations ranging from 0.18 μM to 0.22 μM [8].

Qualitative results were obtained via H&E staining, TEM and confocal microscopy and provided information on morphological changes, microtubule architecture and internal ultrastructures of the SNO cells after exposure to ESE-16. The H&E results revealed the presence of apoptotic morphological characteristics, such as membrane blebbing and apoptotic bodies in the ESE-16-treated. These results were confirmed by studying the internal ultrastructure of the cells via TEM. Results revealed lack of definition of the nuclear membrane, membrane blebbling and apoptotic body formation in the ESE-16-treated cells when compared to the appropriate controls.

Apoptosis occuring in ESE-16-treated SNO cells were studied quantitatively via mitotic indices and the Annexin V-FITC apoptosis-detection assay. Mitotic indices quantified the observed effects in the H&E staining images and revealed a statistically significant increase (*P*-value of 0.0006)^*^ in the percentage of ESE-16-treated cells undergoing apoptosis when compared to the appropriate controls. Results from the Annexin V-FITC apoptosis-detection assay revealed a statistically insignificant increase in MFI (*P*-value of 0.0614) of the ESE-16-treated cells when compared to the appropriate controls. The increased MFI indicates an increase in PS externalization, which is an early apoptotic indicator [35]. The insignificant increase in MFI may be ascribed to the fact that at the time of experimental termination, the cells may have already surpassed the early apoptotic stage. When combined with the qualitative and quantitative results obtained via mitotic indices, H&E staining and TEM, a relationship between ESE-16 exposure and apoptosis can be observed. In addition, the authors have previously demonstrated that the ESE-16 compound induces apoptosis in the esophageal carcinoma cell line with propidium iodide inclusion [32]. In the article by Wolmarans *et al*. cell cycle analysis was performed to study the influence of ESE-16 on the cell cycle progression of the SNO cell line. An increase in the percentage of cells in the sub G_1_ (indicative of apoptosis) in the ESE-16-treated cells was observed when compared to the relevant controls [32].

These results correlates with the findings of Nkandeu *et al*., who tested ESE-16 on MCF-7 breast carcinoma cell line [31]; Theron *et al*., who tested ESE-16 on the tumorigenic human epithelial cervical HeLa cell line [30] and Stander *et al*. who tested ESE-16 on the tumorigenic MCF-7, the metastatic MDA-MB-231 and the non-tumorigenic MCF-12A breast cancer cells [29].

In a comparison of ESE-16 and 2ME’s ability to induce cell death, similarities exist. The effects of 2ME have been studied on various cell lines, including the MCF-7 cell line, the estrogen receptor-negative breast carcinoma cell line MDA-MB-435, the human ovarian adenocarcinoma cell line OVCAR-3 and the renal carcinoma cell line SN12-C [43]. Du *et al*. studied the effects of 2ME on the esophageal carcinoma EC9706 cell line in a time- and dose-dependent study and showed an increase in apoptosis in the 2ME-treated cells after 24 hours at concentrations of 5 μM and 10 μM [7]. Cell death via apoptosis and due to 2ME exposure was also found by Stander *et al*. and Thaver *et al*. when these authors tested the effects of 1 μM 2ME on the MCF-7 breast cancer cell line and the esophageal carcinoma WHCO3 cell line respectively [44, 45]. In comparing results obtained after 24 hours by 1 μM, 5 μM and 10 μM 2ME and 0.2 μM ESE-16 used in this study, the increased potency of ESE-16 can be observed.

ESE-16 has a similar mechanism to that of 2ME, its source compound, and thus also binds to the colchicine binding site situated between the α - and β-dimers of the tubulin protein [8, 9, 14, 17, 18]. It was hypothesized that ESE-16 would, like its source compound, cause abnormal spindle formation and activate the SAC, causing metaphase arrest. This would lead to the inhibition of cell proliferation and cell death. Qualitative and quantitative data obtained in this study indicate that ESE-16 blocks the cells in metaphase.

H&E images revealed hypercondensed chromatin in the ESE-16-treated cells when compared to the appropriate controls. Mitotic indices revealed a significant increase, with a *P*-value of 0.0003^*^, in the percentage of cells in metaphase in the ESE-16-treated samples when compared to the controls. The observed effect of ESE-16 on the microtubules was qualitatively confirmed via confocal microscopy, which also showed hypercondensed chromatin and abnormal spindle formation in the ESE-16-treated cells when compared to the appropriate controls.

2ME has been shown to cause metaphase block in a variety of cell lines (including the MCF-7 cell line, the EC9706 cell line, the WHCO3 cell line and the MDA-MB-435 breast cancer cell line) at concentrations of 1 μM, 2 μM, 5 μM and 10 μM after 24 hours of exposure [7, 19, 44, 45]. The increased potency of ESE-16 can once again be seen, since it was able to cause metaphase block in the SNO cell line after 24 hours’ exposure at a much lower concentration of 0.2 μM.

The mitochondria’s role in the regulation of cell death is well established [40] and is considered to be the central death machinery in the intrinsic apoptotic pathway [35, 46–48], with the key step believed to be mitochondrial membrane permeabilization (MMP) [2, 41, 46–48].

The possible effect that ESE-16 had on the mitochondria of the SNO cells after exposure was studied by means of flow cytometry by quantitatively analyzing ΔΨm and ROS production in the SNO cells after ESE-16 exposure.

Results obtained from studying the ΔΨm showed a statistcally significant (*P*-value of 0.019)^*^ increase in the MFI in the ESE-16-treated cells when compared to the vehicle control. These findings illustrate that ESE-16 caused a decrease in ∆Ψm, which may have led to the degradation of the mitochondrial membrane and apoptosis. Since MMP of the mitochondria is considered a key step in the intrinsic apoptotic pathway [2, 41, 46–48], these results provide evidence of apoptosis taking place via the hypothesized pathway.

The decrease of ∆Ψm due to ESE-16 exposure was also found by Stander *et al*. in the tumorigenic MCF-7 and MDA-MB-231 cell lines. In addition, Stander *et al*. showed that ESE-16 had a more pronounced effect on the tumorigenic cell lines when compared to the non-tumorigenic MCF-12 cell line [29]. The difference in effects shows possible selectivity of ESE-16 toward the more malignant cell lines when compared to non-malignant cell lines.

The mitochondria are both the major source of intracellular ROS and, at the same time, targets of ROS [38, 39, 48]. ROS are natural by-products from the production of ATP, which occurs in the mitochondrial matrix via the oxidative phosphorylation pathway [38, 40, 49, 50]. The accumulation of ROS may lead to oxidative damage to mitochondrial proteins and DNA, causing loss of electron transport, decrease in ATP production and ∆Ψm dissipation [39, 40].

Since O_2_ ^−^ can be regarded as the precursor for most ROS [40], the levels of O_2_ ^−^ were measured via flow cytometry in the SNO cells after ESE-16 exposure. Results revealed an increase in O_2_ ^−^ levels in the ESE-16-treated cells when compared to the relevant controls. This increase shows that ESE-16 causes oxidative stress after 24 hours’ exposure and confirms previously discussed results of ∆Ψm dissipitation, since it is believed that ROS can cause ∆Ψm dissipation [39, 40]. In addition, ROS accumulation is also believed to act as signaling molecules, which may initiate MMP via the permeability transition pore (PTP), causing the release of the pro-apoptotic proteins found in the intermembrane space [39, 51].

Several studies have linked 2ME with the increase in ROS production, specifically O_2_ ^−^[6, 37]. Chua *et al*. showed, however, that 2ME induced only a moderate increase in cellular superoxide while analogues of 2ME extended stronger ROS production, thus suggesting that ROS induction is not a major mechanism of action for 2ME [6]. This correlates with the Stander *et al*. paper of 2010, which showed that 2ME-treated MCF-7 cells had no significant increase in O_2_^−^ levels compared to the vehicle control [44]. By studying various analogues of 2ME, Chua *et al*. suggest that the 2-methoxy group at position 2 in the 2ME compound structure is not required for ROS production and may even have an inhibitory effect [6]. In addition, Chua *et al*. show an increase in O_2_ ^−^ levels due to 2ME analogues that had modifications done on positions 3 and 17 [6]. The increased O_2_ ^−^ production due to ESE-16 might be due to the conformational changes in the ESE-16 compound structure.

After the key step in the intrinsic pathway, believed to be MMP [2, 41, 46–48], several pro-apoptotic proteins, such as AIF and cytochrome *c*, are released into the cytosol [46–48, 52–54]. Once released, cytochrome *c* binds to Apaf-1, allowing deoxyadenosine triphosphate (dATP) to bind onto Apaf-1; inducing conformational changes and causes the oligomerization of Apaf-1 into the Apaf-1 apoptosome [35, 46–48, 53, 54]. This apoptosome subsequently recruits and activates the initiator procasapase 9, which in turn activates downstream effector caspases such as caspase 3, leading to the execution phase of apoptosis [35, 46–48, 53, 54].

Caspase activity in the SNO cells after exposure to ESE-16 was quantitatively studied via spectrophotometry. Results revealed a statistically insignificant (*P*-value of 0.238) increase in caspase 9 activity in ESE-16-treated cells when compared to the relevant controls. In comparison, caspase 3 results showed a statistically significant increase in activity with a *P*-value of 0.004^*^ in the ESE-16-treated cells when compared to the appropriate controls. The difference in the increase of activity of the two caspases might be explained by the fact that the effector caspase 3 had already, at the time of measurement, begun the degradation phase of apoptosis [35, 41] and caused a decrease in caspase 9 levels. The caspase activity results show that ESE-16 causes the activation of caspases and induces cell death in a caspase-dependent manner.

## Conclusion

This was the first study conducted to investigate the action mechanism of the ESE-16 compound on an esophageal carcinoma cell line. In addition to obtaining information of the action mechanism of ESE-16, this study confirmed the increased potency of this compound compared to its source compound, 2ME.

From this study, it can be concluded that the novel *in silico*-designed compound induces cell death at a concentration of 0.2 μM, with an exposure time of 24 hours, in the esophageal carcinoma SNO cell line by disrupting microtubule function. This study unraveled the action mechanism of this novel compound and provides cellular targets for future *in vivo* studies to establish the counpound’s efficacy as a clinically usable anticancer agent. Future studies will investigate the action mechanism of this compound on areas such as angiogenesis; will test whether it exerts any significant side effects and test whether the *in silico*-design has increased the compounds’ bioavailability.

## Materials and methods

### Esophageal carcinoma cell line

SNO esophageal carcinoma cells are non-keratinizing squamous epithelial cells [55] and were purchased from Highveld Biological (Pty) Ltd (Sandringham, SA). The cell line was derived from a well-differentiated squamous cell carcinoma, 6.5 cm in length and metastatic to the lymph nodes, from a Zulu male aged 62 in 1976 [55].

### *In silico*-designed compound

The unique, non-commercially available ESE-16 compound was *in silico*-designed with the use of the Chimera package from the Resource for Biocomputing, Visualization and Informatics at the University of California, San Francisco (supported by NIH P41 RR-01081), which was used for structure preparation and visualization of the compound [8]. Docking studies were carried out with Autodock 4.0 and AutoDockTools4 (Scripps Research Institute, La Jolla, CA, USA) [8]. The ESE-16 compound was synthesized by iThemba Pharmaceuticals (Pty) Ltd (Modderfontein, Gauteng, SA).

### General laboratory reagents and supplies

Dulbecco’s Modified Eagle Medium (DMEM) and F-12 Nutrient Mixture, formulated from single-cell plating of Chinese Hamster Ovary (CHO) cells (HAM’s-F12), was obtained from Sigma-Aldrich Co. (St. Louis, USA). Penicillin, streptomycin, fungizone, gentamycin and trypsin were obtained from Highveld Biological (Pty) Ltd (Sandringham, SA). Phosphate-buffered saline (PBS) was purchased from Gibco-BRL (Invitrogen, Carlsbad, CA, USA). Dimethyl sulphoxide (DMSO), trypan blue, RNase A, Bouin’s fixative and actinomycin D was purchased from Sigma-Aldrich Co. (St Louis, USA). Ethanol was purchased from Merck (Darmstadt, Germany). Fetal calf serum (FCS), sterile cell culture flasks, cell culture plates and syringe filters (0.22 μM) were obtained from Separation Scientific (Randburg, SA). Haematoxylin, eosin, ethanol and xylol were purchased from Merck (Darmstadt, Germany). Alpha-tubulin antibody, alexafluor 488, DAPI and the Mitotracker kit was purchased from Biocom Biotech Pty Ltd. (Clubview, SA). The Annexin V-FITC Apoptosis Detection Kit and the Caspase 3 and - 9 Colorimetric Kits were purchased from BioVision (Mountain View, California, USA).

### Experimental procedures

Experiments were conducted at a concentration of 0.2 μM with a 24-hour seeding time to allow for attachment and a 24-hour exposure time in a humidified atmosphere (37°C with 5% CO_2_). A stock solution of 10 mM ESE-16 was dissolved in DMSO and diluted with medium to the desired concentration prior to exposure of the cells. The concentration of 0.2 μM for ESE-16 was chosen, since previous dose-dependent investigations conducted in our laboratory showed ESE-16 inhibiting cell proliferation to 50% from concentrations ranging from 0.18 μM to 0.22 μM [8].

Experiments were conducted in 6-well plates or 25 cm^2^ cell culture flasks. For 6-well plates, cells were seeded on heat-sterilized coverslips at a density of 5 × 10^5^ cells per well in 3 ml of medium. For 25 cm^2^ cell culture flasks, cells were seeded at 1 × 10^6^ cells in 5 ml of medium.

Appropriate controls were included: cells propagated in complete medium only, a vehicle control that was composed of cells treated with DMSO, the final dilution never exceeding 0.02% (v/v) [15, 56]. Actinomycin D (0.1 μg/ml in growth medium) was used as a positive control for the induction of apoptosis [56].

### Light microscopy

#### Haematoxylin and eosin staining

H&E staining allows for the quantitative comparison of the morphological characteristics of the cytoplasm and nuclear components [10]. Hematoxylin has a deep blue-purple color and stains nucleic acids while eosin is pink and stains proteins nonspecifically [57]. Thus, during a general H&E staining experiment, the nuclei of the cells are stained blue, whereas the cytoplasm and extracellular matrix are stained varying degrees of pink [57]. In order to obtain quantitative data from the morphological study, mitotic indices were determined as described above [10].

SNO cells were seeded in complete growth medium at 5 × 10^5^ cells per well in 6-well plates on heat-sterilized coverslips. Cells were exposed to ESE-16 and the appropriate controls. After the 24-hour incubation period, coverslips were removed from the wells and transferred to a staining dish. Bouin’s fixative was added to the staining dish until the entire sample slide was covered. Samples were incubated at room temperature for 30 min after which the Bouin’s fixative was replaced with 70% ethanol for 20 min to dehydrate the cells. Excess fixative was removed and the staining dish was rinsed with tap water to remove excess ethanol. Haematoxylin was added and the samples were incubated at room temperature for 20 min. The staining dish was rinsed with tap water for 2 min and then rinsed with 70% ethanol to remove any excess stain. Eosin (1%) was added to the staining dish and samples were incubated at room temperature for 2 min. The staining dish was rinsed twice for 5 min with 70% ethanol, twice for 5 min with 96% ethanol, twice for 5 min with 100% ethanol and twice for 5 min with xylol. Coverslips were mounted on microscope slides with resin and left overnight to dry. Samples were evaluated at a magnification of × 20 with Zeiss Axiovert MRc microscope (Zeiss, Oberkochen, Germany).

### Electron microscopy

#### Transmission electron microscopy

TEM allows a beam of coherent electrons to be directed onto a sample under vacuum [58]. Owing to the scattering of electrons within the sample, small objects inside the cell can be viewed directly [58]. This allows for the study and subsequent better understanding of biological structure-function relationships at cellular, subcellular and molecular levels [58–60]. TEM was used to provide precise intracellular information on the SNO cells.

SNO cells were seeded in complete growth medium at 1.5 × 10^6^ cells per 25 cm^2^ flask and were subsequently exposed to ESE-16 and the appropriate controls. After the 24-hour incubation period, cells were trypsinized and the samples were resuspended in 1 ml DMEM complete medium. Samples were fixed with PBS:2.5% gluteraldehyde (9:1) solution for 45 min at room temperature and then rinsed three times for 5 min each with PBS. Samples were fixed again with osmium tetroxide for 15 min and rinsed afterwards three times for 5 min each with PBS. The samples were dehydrated with increasing ethanol concentrations (30%, 50%, 70%, 90%, 100%) and left overnight in 100% ethanol. Samples were then infiltrated and embedded with 100% EMBED 812. Ultrathin sections of the samples were prepared using a microtome and were contrasted using 4% uranyl acetate for 5 min and Reynolds’ lead citrate for 2 min, then rinsed with water and viewed at a scale bar of between 2-10 μm with a Multi-purpose Philips 301 TEM (Electron Microscopy Unit, University of Pretoria, South Africa).

### Confocal microscopy

#### Confocal-alpha (α)-tubulin assay

A confocal microscope creates sharp images by performing point-by-point image construction by focusing a point of light sequentially across a specimen, excluding most of the light from the specimen that is not from the microscope’s focal plane [61]. Confocal microscopy was employed to observe the effects of ESE-16 on the microtubule tubulin dynamics of SNO cells after 24 hours’ exposure time.

SNO cells were seeded in complete growth medium at 5 × 10^5^ cells in 6-well plates on heat-sterilized coverslips and exposed to ESE-16 and the appropriate controls. After the 24-hour incubation period the samples were fixed with 1.5 ml glutaraldehyde fixer for 10 min at 37°C and then permeabilized with 2 ml permeabilization buffer for 15 min at room temperature. Samples were stained first with a 100 μl mouse monoclonal antibody against human α-tubulin (Clone 2-28-33; 1:1000) for 1.5 hours at 37°C. After the incubation period, samples were washed with PBS for 5 min at room temperature and then stained with a secondary antibody (biotin-conjugated anti-mouse IgG 58) in FITC-conjugate diluent (100 μl), for 1.5 hours at 37°C. After three 5 min washes with PBS, coverslips were stained with DAPI (2 ml), a nucleic stain that produces blue fluorescence for 10 min at room temperature. Samples were mounted with a glycerol-based mounting fluid on glass slides and viewed with a Zeiss LSM 510 META confocal laser microscope at the Electron Microscopy Unit at the University of Pretoria (Pretoria, South Africa). Images taken by the microscope were visualized at 10 μm with the use of ZEN 2009 software (Carl Zeiss (Pty) Ltd Johannesburg, South Africa).

### Flow cytometry

#### Apoptosis-detection assay

One of the earliest indications of apoptosis is the translocation of PS from the inner- to the outer leaflet of the plasma membrane where it becomes exposed [8, 62]. Annexin V is a Ca^2+^-dependent phospholipid binding protein with a high affinity for PS [35, 53, 59] and is used when conjugated to a fluorochrome (FITC) as an indicator of early apoptosis [8, 62].

SNO cells were seeded in complete growth medium at 1x10^6^ cells per 25 cm^2^ flasks and were exposed to ESE-16 and the appropriate controls. Samples were trypsinized, resuspended in 1 ml 1x Binding Buffer and centrifuged at 300 × g for 10 min. Supernatant was removed and samples were resuspended in 100 μl of the 1x Binding Buffer. Subsequently, 10 μl of Annexin V-FITC was added and samples were incubated for 15 min in the dark at room temperature. After incubation, samples were washed with 1 ml 1x Binding Buffer and centrifuged at 300 × *g* for 10 min. Supernatant was carefully pipetted off and samples were resuspended in 500 μl 1x Binding Buffer solution. The FL1 channel was used to measure Annexin V-FITC fluorescence and was conducted with an fluorescence-activated cell sorting (FACS) FC500 system flow cytometer (Beckman Coulter South Africa (Pty) Ltd) equipped with an air-cooled argon laser with an excitation wavelength of 488 nm.

### Mitochondrial membrane potential

The Mitotracker kit allows us to measure the ∆Ψm by labelling the mitochondria with a cationic dye named “5,5′,6,6’-tetrachloro-1,133’-tetra-ethylbenzimidazolyl-carbocyanine iodide”, which passively diffuses across the plasma membrane and accumulate in active mitochondria providing red fluorescence [36]. However, if there is a reduction in ∆Ψm, the dye cannot aggregate in the mitochondria and thus remains in the cytoplasm in its monomer form, generating green fluorescence [36].

SNO cells were seeded at 1 × 10^6^ cells per 25 cm^2^ flask and exposed to ESE-16 and the appropriate controls. Samples were trypsinized and centrifuged at 13 000 × g and the supernatant was removed. Samples were resuspended in 1 ml diluted Mitocapture solution and incubated at 37°C for 20 min. Samples were centrifuged at 500 × g, the supernatant was removed and was resuspended in 1 ml pre-warmed (37°C) incubation buffer. Samples were analysed using an FACS FC500 System flow cytometer equipped with an air-cooled argon laser excited at 488 nm (Beckman Coulter South Africa (Pty) Ltd). Apoptotic cells were detected in the FL1 FITC channel showing diffused green fluorescence. Healthy cells were detected in the FL2 channel showing red fluorescence.

### Reactive oxygen species

In the mitochondria, ROS is generated via the electron transport chain and can accumulate as by-products, which can lead to mitochondrial proteins and mitochondrial DNA, causing loss of electron transport, decrease in ATP production and ∆Ψm dissipation [37–40]. In addition, ROS accumulation is believed to act as signaling molecules which initiates MMP, causing the release of pro-apoptotic proteins [39, 51]. Suspected O_2_ ^−^ generation was assessed using HE, an O_2_ ^−^ -sensitive dye that is oxidized by O_2_^−^ to a red fluorescing compound [21, 39].

SNO cells were seeded in complete growth medium at 1 × 10^6^ cells per 25 cm^2^ flask and exposed to ESE-16 and the appropriate controls. Cells were trypsinized and resuspended in 1 ml PBS. Samples were resuspended in 10 μM HE for 15 min at 37°C. Fluorescence was measured on the FL2 channel of an FACS FC500 System flow cytometer (Beckman Coulter South Africa (Pty) Ltd equipped with an air-cooled argon laser with an excitation wavelength of 488 nm.

### Spectrophotometry

#### Caspase activity

The activity of caspase 9 and −3 as a result of ESE-16-exposure was investigated with the use of a Caspase 9 and −3 Colorimetric Kit. The assays were based on the detection of the chromophore *p*-nitroanilide (*p*NA) at a wavelength of 405 nm via spectrophotometry.

SNO cells were seeded in complete growth medium at 1 × 10^6^ cells per 25 cm^2^ flask and exposed to ESE-16 and the appropriate controls. Cells were trypsinized and centrifuged at 250 × g for 10 min. Supernatant was removed and samples were lysed with the addition of cold lysis buffer and were incubated on ice for 10 min. Samples were centrifuged at 10 000 × g for 1 min and supernatant was transferred to new tubes and kept on ice. This was expected to yield a cell lysate with an approximate protein concentration of 2–4 mg/ml. Cell lysate (50 μl) were added to wells in a 96-well plate. 2× Reaction Buffer (50 μl) containing dithiothreitol (DDT) stock was added to samples along with 5 μl of caspase-3/-9 colorimetric substrate. The plate was incubated at 37°C for 1–2 hours. Absorbance was determined at a wavelength of 405 nm with the use of an EL_x_800 Universal Microplate Reader (Bio-Tek Instruments Inc. Vermont, USA).

### Statistical analysis

Qualitative analysis was obtained via light microscopy, confocal microscopy and TEM. Quantitative analysis was obtained via mitotic indices, flow cytometry and spectrophotometry. All experiments conducted were repeated three times. For the techniques that involved flow cytometry, 10 000 to 30 000 events were counted for each repeat and analysis of the data was done with the use of the Cyflogic program, version 1.2.1, created by Perttu Terho and Mika Korkeamäki from CyFlo Ltd. based in Finland. Quantitative data were analysed for significance by using student *t*-test statistics. A *P*-value of <0.05 was regarded as statistically significant.

## Abbreviations

2ME: 2-Methoxyestradiol
∆Ψm: Mitochondrial membrane potential
Apaf-1: Apoptotic protease activating factor 1
ATP: Adenosine triphosphate
CAII: Carbonic anhydrase II
CAIX: Carbonic anhydrase IX
CHO: Chinese Hamster Ovary
DAPI: 4’,6-Diamidino-2-phenylindole
dATP: Deoxyadenosine triphosphate
DDT: Dithiothreitol
DMEM: Dulbecco’s modified eagle medium
DMSO: Dimethyl sulphoxide
DNA: Deoxyribonucleic acid
EA: Esophageal adenocarcinoma
EC: Esophageal cancer
ESCC: Esophageal squamous cell carcinoma
ESE-16: 2-Ethyl-3-*O*-sulphamoyl-estra-1,3,5(10)16-tetraene
FACS: Fluorescence-activated cell sorting
FCS: Fetal calf serum
FITC: Fluorescein isothiocyanate
H&E: Haematoxylin and eosin
MFI: Mean fluorescent intensity
MMP: Mitochondrial membrane permeabilization
MIDs: Microtubule-interfering drugs
NAD^+^: Nicotinamide adenine dinucleotide
NADH: Reduced nicotinamide adenine dinucleotide
NADP^+^: Nicotinamide adenine dinucleotide phosphate
NADPH: Reduced nicotinamide adenine dinucleotide phosphate
NCD: Nanocrystal dispersion
O_2_ ^−^: Superoxide
PBS: Phosphate buffered saline
PS: Phosphatidylserine
PTP: Permeability transition pore
ROS: Reactive oxygen species
SAC: Spindle assembly checkpoint
TEM: Transmission electron microscopy.
